# Supplementary figures and images for: Dual mechanisms of opioid-induced respiratory depression in the inspiratory rhythm-generating network
Source: eLife. 2021 Aug 17;10:e67523. doi: 10.7554/eLife.67523 (PMC8390004; doi:10.7554/eLife.67523)

**
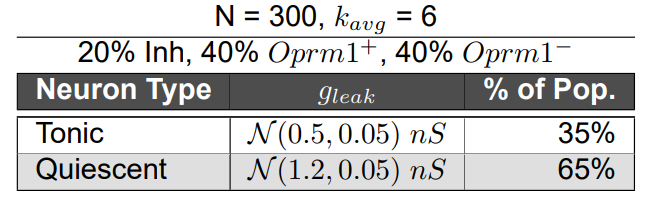
**

**Table 1:** Network parameters for *in silico* preBӧtC network

Supplement: Supplementary file 1. [file elife-67523-supp1.docx]

**
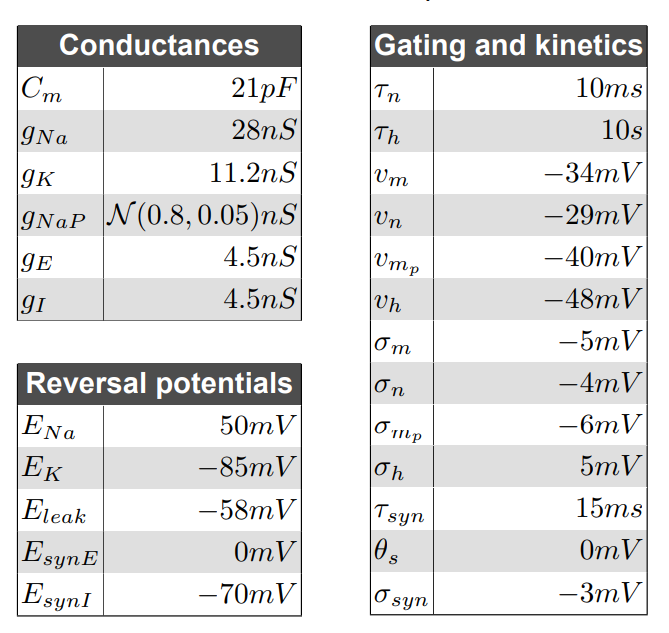
**

**Table 2:** Cellular parameters for *in silico* preBӧtC neurons

Supplement: Supplementary file 2. [file elife-67523-supp2.docx]
